# Impact of work experience placements on school students’ attitude towards mental illness

**DOI:** 10.1192/pb.bp.114.046714

**Published:** 2014-08

**Authors:** Vanathi Kennedy, Ravindra B. Belgamwar

**Affiliations:** 1 Birmingham and Solihull Mental Health NHS Trust, Birmingham; 2 North Staffordshire Combined Healthcare NHS Trust, Stoke-on-Trent

## Abstract

**Aims and method** Research shows that 16- to 19-year-olds express the greatest level of negative attitudes towards people with mental illness. Our aim was to assess the effectiveness of work experience placements in influencing secondary-school students’ attitudes towards mental illness and career choices. The Adolescent Attitude Towards Mental Illness questionnaire measured and assessed the adolescents’ attitude changes. Pre- and post-evaluation questionnaires assessed changes in their career choices.

**Results** There was a statistically significant change in the adolescents’ attitudes, especially regarding categorical thinking and perceptions that people with mental illness are violent and out of control. There was also a positive shift in their career choices towards options in the field of mental health.

**Clinical implications** Work experience placements can have a positive impact on secondary-school students’ attitudes towards mental illness and may improve the level of student recruitment into the field of psychiatry.

The use of the word ‘stigma’ occurred as early as ancient Greece, even before the term ‘psychiatry’ was used. The word ‘stigma’ was derived from the term ‘stigmata’, which means a physical mark of disgrace. Later, with time, the term ‘stigma’ became attributed to the shame and dishonour related to one’s social characteristics.^1^ The stigma associated with mental illness has a huge negative impact on several aspects of the lives of those with a mental illness. Numerous studies have discussed the negative impact of this stigma on the psychological well-being of patients^2,3^ as well as on their self-esteem^2,4-6^ and their social and occupational opportunities.^7^ A study conducted by representatives from the ‘Time to Change’ campaign demonstrated that nine out of ten people with mental health problems (87%) reported the negative impact of stigma and discrimination in their lives.^8^ Given the negative impact of stigmatisation, there have been a number of studies on ways to combat the stigma surrounding mental illness. Corrigan & Watson^9^ illustrated the three main strategies: protest, education and contact. Although protest seemed to have some effect in reducing negative attitudes, it did not influence any positive attitudes. However, the strategies of education and contact have demonstrated the ability to enhance positive attitudes towards people with mental illness.^10^ Allport’s (1954) ‘contact hypothesis’ theory for social relations was later expanded to assist with combating mental health stigma (p. 537).^11^ In addition, a number of methods have been used to study the impact of contact in influencing attitude towards mental illness.^2,12-15^ Research has determined that the stigma attached to mental illness is mainly a result of the misconceptions others have that people with mental illness are dangerous and unpredictable. This stigmatisation stems from a fear of the unknown. People with mental illness have repeatedly conveyed that the stigma and discrimination frightens them more than the worst symptoms of their illness. Research has illustrated how this fear of stigma creates a process of self-stigma and acts as a major barrier to seeking help and treatment for people with mental illness.^16,17^

A number of anti-stigma campaigns and other interventions have been undertaken by the Royal College of Psychiatrists and various volunteer organisations in the UK in order to alter public attitudes towards mental illness, as well as to enhance the quality of life and experience of people with mental illness.^18^ Most of the campaigns are targeted at adult populations. Not much is known about young people’s understanding of mental illness and about how they view people with mental illness. A follow-up study of the ‘Changing Minds’ campaign, organised by the Royal College of Psychiatrists, found that ‘the greatest proportion of negative opinions about mental illness was in the 16-19 year age group, and respondents with higher education were less likely than the rest to express such views’.^19^ The stigma surrounding mental illness does not emerge unpredictably after adolescence. The thoughts and perceptions that influence stigmatisation develop from childhood and evolve throughout adulthood. Adolescence is a stage when individuals are rapidly maturing, both physically and emotionally, and this would be the best period to target their attitudes, while those attitudes can still be moulded. Hence it is important that combating mental health stigmatisation be instigated in early childhood so it becomes a part of children’s normal social development.^20,21^ The College has recommended that mental health trusts organise work experience placements for secondary-school students in an attempt to influence the students’ attitudes and improve recruitment into psychiatry.

Another major problem facing psychiatry is poor recruitment of new students and practitioners into the field, a problem that has persisted for the past 40 years. Brockington & Mumford^22^ reported that the number of medical students choosing psychiatry as a career remained stable at about 3.6%. The *F2 Career Destination Survey (2012)* suggests that only 2.8% of UK trained graduates and 3.1% of foundation year 2 (FY2) doctors opted to pursue careers in psychiatry.^23^ There was significant variation that was noted (for example 5.9% of students from the Dundee Medical School and 8.2% of Staffordshire FY2 doctors chose to pursue psychiatry, compared with only 1.6% from the Cambridge School of Medicine and 0.7% of Black Country FY2 doctors). There are a number of programmes organised for medical students and foundation doctors to improve recruitment into the field of psychiatry. However, there is very limited work being done in an effort to develop the interest of younger individuals. A programme that provides work experience placements to secondary-school students was organised by the North Staffordshire Combined Healthcare NHS Trust. The primary aim was to provide education and awareness about the NHS, mental healthcare and a range of career opportunities in the healthcare sector. The secondary aim was to assess the effectiveness of the work experience programme in influencing adolescents’ attitudes towards mental illness and career choices in the field of psychiatry.

## Method

When looking into the limited available literature on strategies and interventions to influence adolescent attitudes towards mental illness, it was evident that education, contact or a combination of both were needed in order to be effective in improving knowledge and influencing attitudes and intended behaviours.^9,24^ As a result of these findings, we planned to organise a structured programme that would provide a holistic experience, including contact with people with mental illness, as well as an educational component designed to improve adolescents’ awareness of the areas of mental health, functions of the NHS and career options available in the field of mental healthcare. Our goal was to provide an opportunity for local high-school students to attend work experience placement at a local psychiatric hospital.

The programme’s design included 4 days of work experience placements for two cohorts per year. One programme took place in April 2012 for GCSE students and the other occurred in July 2012 for ‘A’ level students. Invitations were sent to all secondary schools in Stoke on Trent and the North Staffordshire area. A total of 32 students participated in the placement programme.

### Contents of the programme

The programme was designed to be interactive and stimulating for the students. The students had their induction on the first day, with an interactive lecture on the NHS and a mental health quiz. The lecture and quiz were focused on the functions of the NHS and mental health trusts, various settings in mental health hospitals, different mental health conditions and the treatments available. An interactive session on ‘Stigma and mental health’ and a talk by a previous work experience student on how to make the most of the placement were provided, both of which were highly appreciated by the students.

Students then met with various professionals at the career market, where the students and professionals participated in an activity similar to a ‘speed dating’ session. During this session, students had the opportunity to meet with different members of the multidisciplinary team, such as doctors, psychologists, social workers, occupational therapists, psychiatric nurses, physiotherapists, administrative staff members and hospital managers to discuss career options available within the field of mental health. This was followed by an interactive mannequin session on physical examination and phlebotomy, as well as a session on how talking therapy (i.e. cognitive-behavioural therapy) works. They then had a tour of the hospital, including the electroconvulsive therapy suite. On the second and fourth days, students spent time with their chosen healthcare professionals, where they had contact with patients in various settings, such as out-patient clinics, domiciliary visits, therapy sessions. On the third day, students visited the Keele Medical School, where they attended an anatomy lab session, facilitated by the lecturers, which gave them a feel for a medical student’s life. The students also had lectures on medical ethics and law.

### Measures

The Adolescent Attitude Towards Serious Mental Illness (ATSMI-AV) questionnaire was used to measure and assess the attitude changes of the students.^20^ The students were asked to complete the questionnaire both before and after the placement. The ATSMI-AV is a 21-item validated scale developed to measure various attitudes about mental illness that are relevant to adolescents. It is a measure that includes perceptions of violence, social avoidance, embarrassment if diagnosed as having a mental illness, and personal vulnerability to mental illness.^18,20^ The responses to each item are scored using a five-point scale (from 1, indicating ‘strongly disagree’ to 5, indicating ‘strongly agree’).

Watson *et al*,^20,25^ who devised this questionnaire, categorised the 21 items on this scale into five different factors, depending on the various components of attitude that the items were meant to measure. The factors were: ‘threat’, ‘social concern’, ‘wishful thinking’, ‘categorical thinking’ and ‘out of control’. Factor 1 had six questions, Factor 2 had five questions, Factor 3 had four questions, Factor 4 had four questions and Factor 5 had two questions.

The six questions in Factor 1 reflect the fear of harm from people with mental illness and the feeling of embarrassment of having mental illness (reputation), hence the classification name, ‘threat’. The five questions in Factor 2 replicate the fear of mental illness being used as a label to control people and the fear of getting a mental illness, hence the classification of ‘social control/concern’. Factor 3’s four questions represent the idea that people with mental illness can be cured if they tried hard enough and that they could be cured if they are treated with love and kindness. This is termed ‘wishful thinking’. Although the thought of treating people with mental illness with kindness and being optimistic about their prognosis is welcomed, it is important for people to have realistic knowledge about mental illness. Unrealistic perceptions can lead to adding pressures on people with mental illness, expecting them to try hard to get better and imparting the idea that they are lazy. The four questions in Factor 4 reflect the concept that ‘one’s life is pretty much over if they get a mental illness’, hence the term ‘categorical thinking’. The two questions in Factor 5 suggest that people with mental illness get the mental illness because they choose to do bad things.

The statistical analysis was done using a Wilcoxon matched pairs test, as it is one of the statistical tests used to analyse data of repeated measures on the same participants.

To assess the effectiveness of the programme and its impact on the students’ career choices, we designed a questionnaire, which was completed at the beginning of the programme. The questionnaire had questions regarding intended career choices, expectations of the placement and what respondents desired to achieve from this placement. On the last day, the students were given an evaluation form in order to get information about the positives and negatives of the placement, to assess how well the placement met their expectations, and to identify their preferred career choices after completing the placement. As the questionnaire was devised by us and was not validated, statistical analysis was not done. We have presented the results in numerical values.

## Results

A total of 32 students (16 GCSE and 16 ‘A’ level students) completed the pre-placement questionnaire and 24 students (15 GCSE and 9 ‘A’ level) completed the post-placement questionnaire. Of the 32 students, 81% (*n* = 20) were female, 13% (*n* = 4) were Asian-British, 9% (*n* = 3) were African-Caribbean-British, and 78% (*n* = 25) were White British.

The mean and standard deviation were calculated for each factor. Higher scores indicated more negative attitudes and lesser scores indicated positive attitudes. For example, for the first factor, ‘threat’, there were six questions. The responses were rated 5-1, and thus the maximum score would be 30 and the minimum score would be 5. The mean for each factor was calculated by adding the scores of each question in that factor and dividing by the number of questions. Hence, for Factor 1, if the added scores of the six questions were 16, the mean was 2.66. The same students were measured on both occasions, and thus the data was ‘paired.’ As only 24 students completed the post-placement questionnaire, analysis was done for the 24-paired data. As a result of the small sample size, the analysis was done using a Wilcoxon matched-pairs test. For this test, a sample size of ten is good enough for a reliable result.^26^

**Table 1:** Mean and standard deviation of the pre- and post-placement questionnaire values for the factor-based scores^a^ (*n* = 24)

Factor	Pre-placement, mean (s.d.)	Post-placement, mean (s.d.)	*P*
Threat	2.54 (0.48)	1.94 (0.30)	< **0.001**
			
Social concern	2.04 (0.32)	1.82 (0.42)	0.05
			
Wishful thinking	3.04 (0.57)	2.92 (0.67)	0.72
			
Categorical thinking	2.33 (0.67)	1.73 (0.32)	< **0.001**
			
Out of control	2.96 (0.51)	2.42 (0.80)	**0.007**

Results in bold are significant.

a. Factor-based scores range from 1 to 5.

The results in Table 1 suggest that there were statistically significant differences between the pre- and post-test values for Factors 1, 4 and 5. For each of these three factors, the post-test values were significantly lower than the pre-test values. For example, the mean for Factor 1 reduced from 2.5 to 1.9 between the two tests. There was also some evidence of a reduction in values for Factor 2, but this result was only of borderline statistical significance (*P* = 0.05). The values of Factor 3 did not vary significantly between the two time points. The results suggest that work experience placements for secondary school children can favourably influence their categorical thinking about mental health and perceptions of people with mental illness.

### Career intentions

**Fig 1:**
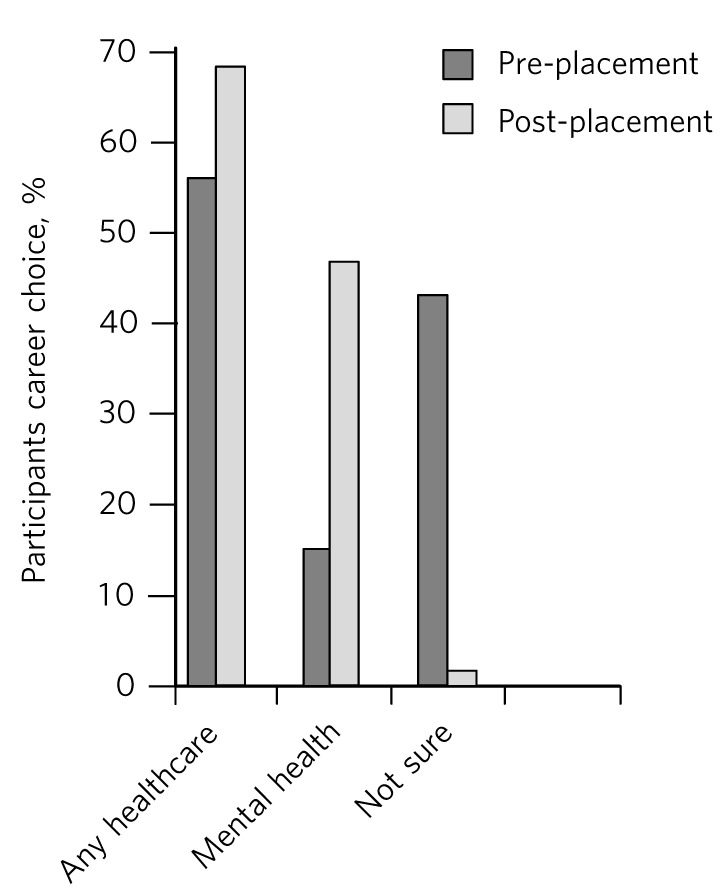
Career choices before and after placement.

We used self-reports on career intentions before and after the placement to determine a change. With regards to influencing the students’ career choices, there was a significant change towards choosing a career in mental health (Fig. 1). There were 32 students who completed the pre-placement questionnaire, whereas only 24 of them returned the post-placement questionnaires. At the start of the placement, 56% (*n* = 18) of the students stated that they were planning to pursue a career in healthcare and wished to attend the placement to get more information about the NHS and career options available in healthcare. However, 92% (*n* = 22) definitely wished to pursue a career in healthcare following the placement.

Although 56% (*n* = 18) of the students originally wished to pursue a career in healthcare, only 16% (*n* = 5) of the students wished to pursue a career in mental health preplacement, and this number significantly increased to 63% (*n* = 15) post-placement. Of the 44% (*n* = 14) of the students who were not sure of their career choices at the time of starting the placement, 92% (*n* = 13) seemed to have had a positive experience during the placement and wished to pursue a career in healthcare following the programme. There were 8% (*n* = 2) who continued to be indecisive about their career options following the placement.

There were 22 students out of 24 who completed the post-placement evaluations that were very appreciative, stating that the placement gave them an overall understanding about the functioning of the NHS and about mental health. They also mentioned that they had imagined a mental health hospital to be the old asylum stereotype and that they were amazed to see a state-of-the-art building and a friendly atmosphere.

## Discussion

There are a number of studies that look at interventions and strategies used to influence attitudes towards mental illness among members of the adult population. There have also been studies that explored ways to improve recruitment into the field of psychiatry for medical students and foundation doctors, including establishing psychiatric summer schools. However, there are not many studies that focus on identifying strategies to influence attitudes of younger adults and whether those strategies might improve recruitment into careers available in the field of psychiatry. Adolescence is an age when people are actively experimenting with their life experiences in an attempt to understand the world in general. Strategies to enhance adolescents’ understanding of mental illness early on in their life can go a long way in preventing the misconceptions and misperceptions about mental illness, which in turn can impart a positive influence on them. The few research studies done so far involving young people looked into the effectiveness of providing mental health awareness programmes by means of utilising lectures, discussions on case vignettes related to mental illness, experiences of service users and demonstrations affirming positive influences.^26,27,29^ Whereas in our work experience placement, we provided adolescents with first-hand experience of seeing a psychiatric hospital and the opportunity to talk with patients in the out-patient clinics. The results of the study were positive in favourably influencing the adolescents’ attitudes regarding the three factors, ‘threat’, ‘categorical thinking’ and ‘out of control’. There was also a trend towards positive change with regards to the factor termed ‘social control’. With respect to the factor termed ‘wishful thinking’, the results point towards a negative attitude change. This could have been addressed by providing a more in-depth knowledge of the treatment and prognosis of different mental health programmes. We can only infer that a 4-day placement would be too short a period of time to provide the participants with in-depth information.

Overall, our findings show that even short work experience placements can help in educating school children and improving their awareness about mental health and their attitudes towards mental illness. Work experience placements can target adolescents’ perceptions about people with mental health problems. Our sample size was small, although 32 seems to be a good enough number for the statistical test used to give a reliable result. A larger sample size would have given a statistically significant difference for the second factor, as the results show a trend towards positive change. However, for practical reasons we could not have a larger number of students at one time. (The sample size of above ten is considered a good enough number for a reliable result when using Wilcoxon’s sign rank test for statistical analysis).^26^

### Limitations

One limitation was a small sample size. With a larger sample size, responses for some questions where there was a trend towards positive changes might have shown a statistically significant change. Another limitation was that there was no control group to compare the results with and randomise the students. There was also no standardisation of the programme between the two cohorts (A level and GCSE students). Another limitation is that the self-report questionnaire used for assessing career intentions has not been validated. There is a chance of social desirability bias, as it is notable that even the scores at the pre-placement stage did not show much of a negative attitude. The mean scores were around two. Even then, the results show that the placement has further shifted students’ attitudes in a more positive direction. It is debatable as to the persistence of the attitude changes over time, and whether it would be ideal to provide the students with booster sessions. More research is needed to look at the persistence of change and ways of enhancing those changes.

### Implications

Work experience placements seemed to influence the students’ career choices towards careers in the mental health field. Even for students who preferred other areas in the healthcare field, the placement was an eye-opener for them, increasing awareness of the psychological impact of medical problems. The placement received very positive feedback from students, teachers and parents. It appears that the ‘contact hypothesis’ is very relevant in reducing stigma, particularly in young people, and we hope that our experience will encourage others to run similar programmes in their area. The Royal College of Psychiatrists has produced guidance on work experience placement, which is available on the college website.^30^
